# Supplementary material for: Influence of the Carrier Gas Flow in the CVD Synthesis of 2-Dimensional MoS2 Based on the Spin-Coating of Liquid Molybdenum Precursors
Source: Nanomaterials (Basel). 2024 Oct 31;14(21):1749. doi: 10.3390/nano14211749 (PMC11547744; doi:10.3390/nano14211749)
Supplement: Supplementary file 1 [file nanomaterials-14-01749-s001.zip › nanomaterials-3262540-supplementary.pdf]

Supporting Information

Influence of the carrier gas flow in the CVD synthesis of 2-dimensional MoS_2 based on the spin-coating of liquid molybdenum precursors.

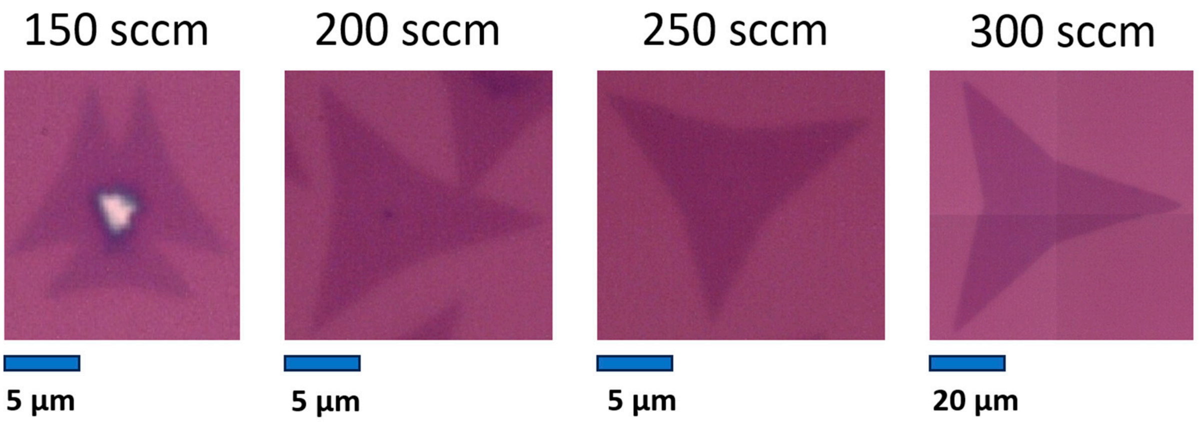

Figure S1: *OM images of the flakes analyzed by Raman maps.*

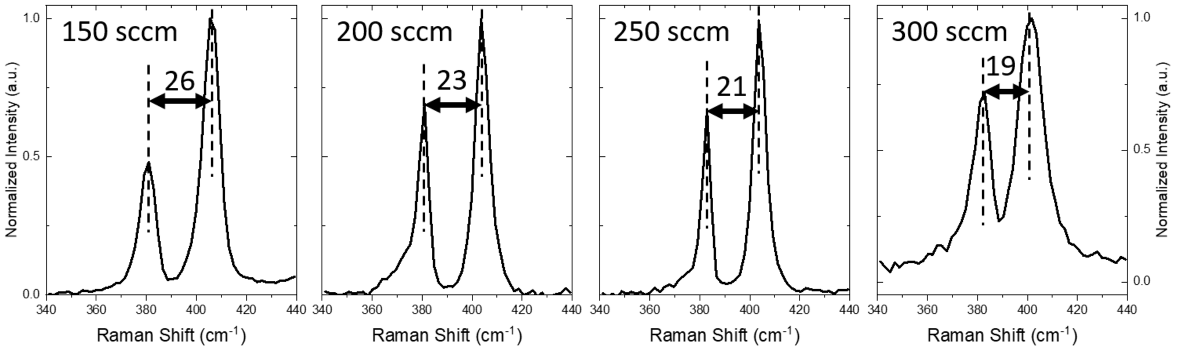

Figure S2: *Representative Raman spectra of MoS_2 for 150-200-250-300 sccm growth processes, collected in the central part of the flake. The spectra reported the Raman separation mode. The Raman spectrum of the 150 sccm specimen is collected on the central bulk-like part of the structure.*

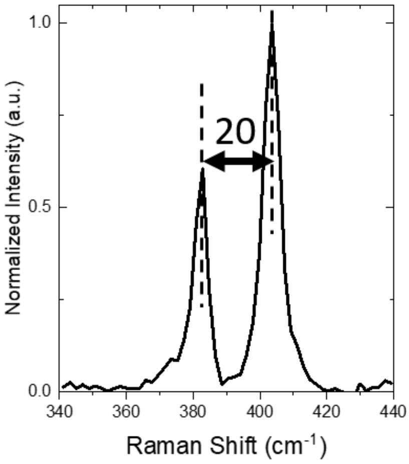

Figure S3: *Representative Raman spectrum of MoS_2 for 150 sccm growth process, collected in the lateral part of the structure.*
